# Effect of Ankle Plantar Flexor Spasticity Level on Balance in Patients With Stroke: Protocol for a Cross-Sectional Study

**DOI:** 10.2196/16045

**Published:** 2020-08-21

**Authors:** Ashraf Mahmoudzadeh, Noureddin Nakhostin Ansari, Soofia Naghdi, Ebrahim Sadeghi-Demneh, Omid Motamedzadeh, Brandon S Shaw, Ardalan Shariat, Ina Shaw

**Affiliations:** 1 Department of Physiotherapy School of Rehabilitation Tehran University of Medical Sciences Tehran Iran; 2 Neuroscience Institute Sports Medicine Research Center Tehran University of Medical Sciences Tehran Iran; 3 Prosthetics and Orthotics Department Musculoskeletal Research Center Isfahan University of Medical Sciences Isfahan Iran; 4 Department of Human Movement Science University of Zululand Kwazulu-Natal South Africa

**Keywords:** stroke, muscle spasticity, balance, rehabilitation, lower extremity, posturography

## Abstract

**Background:**

The lower limb spasticity after stroke can affect the balance and gait of patients with stroke.

**Objective:**

The aim of this study is to assess the effects of ankle plantar flexor spasticity level on balance in patients with stroke.

**Methods:**

Patients with stroke were recruited from neurology and physiotherapy clinics in Tehran, Iran. Based on the level of ankle plantar flexor spasticity according to the Modified Modified Ashworth Scale (MMAS), the eligible patients with stroke were divided into 2 groups: high spasticity (MMAS score≥2) and low spasticity (MMAS score<2). The primary outcome measures were the MMAS scores, Activities-Specific Balance Confidence questionnaire scores, eyes-open and eyes-closed posturography measures, and Timed Up and Go test results. The secondary outcome measures were the ankle passive range of motion and ankle joint proprioception. The t test, mixed model univariate analysis of variance, and Spearman rank correlation were used for statistical analysis.

**Results:**

Data collection and statistical analysis are complete. The interpretation of results is underway. We expect the results to be published in winter 2020.

**Conclusions:**

We believe that patients with high ankle plantar flexor spasticity after stroke will demonstrate greater balance dysfunction, which will worsen with impaired proprioception, passive range of motion, and eyes closed.

**International Registered Report Identifier (IRRID):**

RR1-10.2196/16045

## Introduction

Stroke is the most common cause of disability in adults worldwide. Spasticity is one of the most important motor complications after stroke and negatively affects patients’ quality of life [1,2]. Spasticity is a velocity-dependent increase in muscle tone, resulting from hyperexcitability of the stretch reflex [3]. The lower limb spasticity has a critical role in balance and gait dysfunction of patients after stroke [4]. It decreases the joint range of motion (ROM) and increases the stiffness of the muscles and tissues around the joints. The impairment in balance and postural control is an important symptom in patients after stroke, because it can delay the recovery process in performing daily activities and increases the risk of falling [5]. A reduced balance control is associated with greater disability [6].

The somatosensory system, especially proprioception, is impaired in patients with stroke [7]. This impairment affects the motor function of the patients and prolongs their rehabilitation period. Consequently, the balance control is difficult for the patients with stroke due to impaired proprioception and inappropriate ankle strategies [8].

The evaluation of balance and of the factors contributing to the balance disorders, such as balance nonconfidence, in patients after stroke is necessary. The balance confidence indicates the patients’ confidence to maintain their balance and stability. Balance nonconfidence can affect both static as well as dynamic balance and subsequently increases the chance of falling and disability. Decrease of static and dynamic balance is a significant risk factor of falling and a functional limitation of daily activity [9,10]. Balance has a direct relationship with functions such as walking and climbing the stairs [11]. Balance in patients with stroke is the key factor in the prediction of rehabilitation period and functional outcomes [12].

The lower limb spasticity can affect the gait quality and balance of patients after stroke [13]. The role of spasticity in falling and the direct relationship between the severity of spasticity and the history of falling have been demonstrated [14,15]. Rahimzadeh Khiabani et al [16] evaluated the relationship between spasticity severity and balance in patients with stroke. However, this study had several drawbacks. The severity of spasticity was measured based on the Modified Ashworth Scale (MAS), despite the debate on the scale’s reliability and validity [17] and the caution against its use for assessing spasticity [18]. Furthermore, only static balance, not proprioception and ankle ROM, was evaluated. Therefore, the main objective of this study protocol is to investigate the effects of ankle plantar flexor spasticity level on the balance of patients with stroke. We hypothesized that the patients with high level of ankle plantar flexor spasticity have greater balance dysfunctions, especially in the eyes-closed condition, and that their balance confidence is lower than that of the patients with a low level of spasticity in the eyes-open condition.

There are no optimal tools for assessing balance in patients with stroke. This study assessed the balance using valid clinical tools and instrumented posturography, as the objective measurement of balance is important to detect dysfunctions. Instrumented posturography that uses a force plate is inexpensive and easily available. Therefore, it was used to quantify postural sways through the measurement of center-of-pressure displacements during quiet standing. Balance dysfunctions in the patients with stroke are frequently characterized by deviations and instability of the center of pressure. Therefore, using the instrumented posturography for assessing the static balance is relevant.

## Methods

### Study Design

A cross-sectional study was designed to compare the static as well as dynamic balance, balance confidence, ankle proprioception, and passive ROM between 2 groups of patients with the high and low levels of ankle plantar flexor spasticity after stroke.

### Setting

The measurements were be taken at the Biomechanics and Analysis of Human Motion Laboratory, School of Rehabilitation, Tehran University of Medical Sciences in Iran.

### Approval of Study Protocol

The study protocol was approved by the Review Board, School of Rehabilitation, Tehran University of Medical Sciences and the Ethics Committee of Tehran University of Medical Sciences (Reference number: IR.TUMS.FNM.REC.1397.012).

### Informed Consent

All eligible participants provided a written formal consent after receiving information about the research procedure. We explained the study details to participants before taking the measurements.

### Participants

Participants with stroke were recruited from the neurology and physiotherapy clinics in Tehran, Iran. Participants were screened for eligibility. The patients were divided into 2 groups based on their level of ankle plantar flexor spasticity according to the Modified Modified Ashworth Scale (MMAS): high spasticity (MMAS score≥2) and low spasticity (MMAS score<2).

The inclusion criteria were as follows: first-ever unilateral stroke (hemorrhagic/ischemic), ankle plantar flexor spasticity≥1 based on the MMAS, walking ability, no fixed contracture in the ankle, independent standing with eyes open/closed, ability to understand and follow the commands, and no pain in the lower limbs. Participants with vision problems or depression as well as those taking antispastic medications or undergoing a rehabilitation program focused on balance and proprioception were excluded.

The physiotherapy and neurology clinics in Tehran were contacted for referring the patients with stroke who were willing to participate in the study. The principal investigator and physiotherapist responsible for assessing the patients and performing the experiments called the heads of these clinics to request cooperation and to describe the eligibility criteria. Moreover, the study aims and eligibility criteria for inclusion of patients were provided in the written form to the heads of the clinics.

### Sample Size

Considering the data from the previous study [16], the sample size was estimated to be 28 (n=14 in each group; Z_α_=1.96; α=.05; Z_β_=.842; standard effect size=1.067).

### Procedures

**Figure 1:**
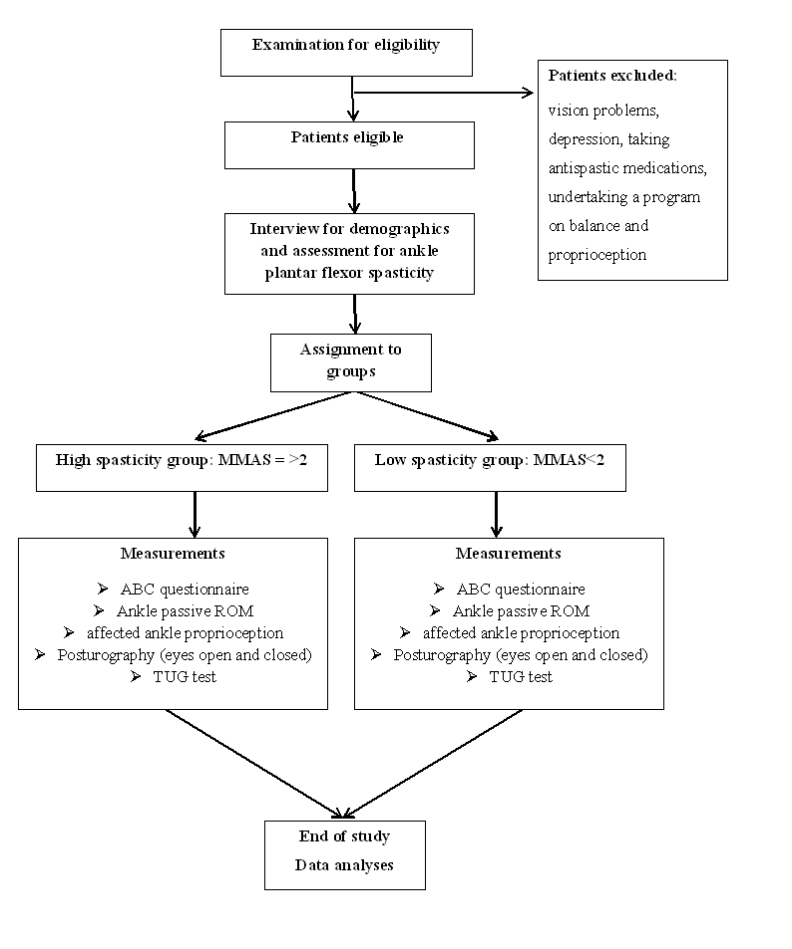
Representation of the study protocol. ABC: Activities-Specific Balance Confidence; ROM: range of motion; TUG: Timed Up and Go.

The patients were interviewed to collect demographic data, including age, gender, height, weight, time since the onset of stroke, etiology (ie, ischemic or hemorrhagic), and the affected side. Patients were assigned to one of the following groups: high spasticity (MMAS score≥2) and low spasticity (MMAS score<2). The severity of ankle plantar flexor spasticity was measured after making the patients rest in bed for 5 minutes in supine position with their shoes taken off [19]. Subsequently, the Activities-Specific Balance Confidence (ABC) questionnaire [20] was administered, followed by the measurements of affected ankle proprioception, passive ROM, posturography, and Timed Up and Go (TUG) test (Figure 1). An experienced physiotherapist performed all the tests.

### Outcome Measures

The primary outcome measures were the MMAS scores, ABC questionnaire, posturography measures in open- and closed-eyes conditions, and TUG test. The secondary outcome measures were the ankle passive ROM and ankle joint proprioception. Table 1 summarizes the outcomes and how they were measured in the study.

**Table 1:** Summary of the outcome measures.

Outcomes		Scale of measurement
**Primary outcomes**		
	Spasticity	MMAS^a^
	Balance confidence	ABC^b^ questionnaire
	Static balance	Posturography with eyes open and closed
	Dynamic balance	TUG^c^ test
**Secondary outcomes**		
	Passive ROM^d^	Standard goniometer
	Ankle proprioception	Electrogoniometer

^a^MMAS: Modified Modified Ashworth Scale.

^b^ABC: Activities-Specific Balance Confidence.

^c^TUG: Timed Up and Go.

^d^ROM: range of motion.

### Spasticity

The affected ankle plantar flexor spasticity was assessed by an experienced physiotherapist using the reliable and valid MMAS [21,22]. To assess the spasticity severity, the physiotherapist stood on the affected side, stabilized the affected ankle with one hand, and moved it from maximum possible plantar flexion to maximum possible dorsiflexion, counting to 1001 [23]. The definitions of spasticity grades of MMAS are presented in Table 2.

**Table 2:** Modified Modified Ashworth Scale (MMAS) to assess the level of spasticity [17].

Grades	Definitions
0	No increase in muscle tone.
1	Slight increase in muscle tone, manifested by a catch-and-release or by minimal resistance at the end of the ROM^a^, when the affected part(s) is moved in flexion or extension.
2	Marked increase in muscle tone, manifested by a catch in the middle range and resistance throughout the remainder of the ROM, but affected part(s) is easily moved.
3	Considerable increase in muscle tone; passive movement is difficult.
4	Affected part(s) is rigid in flexion or extension.

^a^ROM: range of motion.

### Balance Confidence

The ABC questionnaire, which is reliable and valid, was used to assess the balance confidence of patients with stroke in performing their daily activities [20,24]. The ABC questionnaire included 16 questions asking the subjects to score their confidence from 0% (no confidence) to 100% (complete confidence). To calculate the total score in percent, the following formula was used: (total score/16)×100.

### Posturography

The static balance of patients was evaluated by posturography. The use of force plate in balance measurement of the patients with stroke has been demonstrated [25]. The physiotherapist asked each patient to stand on the force plate with bare feet, heels apart by 9 cm and at 30° angle, and upper limbs comfortably along the body. The patients were asked to look at a point on the wall at a distance of 2 m during the test with eyes open as well as with eyes closed. Open- or closed-eyes condition was randomly applied with 2-minute rest interval between the conditions. Each condition was repeated 3 times (with a 20-second interval), and the duration of each repetition was 20 seconds. Velocity (in centimeters per second) and the anteroposterior and mediolateral displacements (in centimeters) were recorded 3 times, and an average was calculated [25].

### TUG Test

Dynamic balance of patients was measured by TUG Test, which has been proven reliable in patients with stroke [26]. The patient was asked to sit comfortably on the chair with feet resting on the floor. Then the patient was asked to get up from the chair, walk a 3-meter distance, turn around, go back to the same chair, and sit down. The time in seconds was recorded using a stopwatch from the moment the patient got up from the chair to the moment he or she sat back on the chair.

### Ankle ROM

The ankle passive ROM in degree was measured in the supine position with knee extended using a standard goniometer. Axis of the goniometer was located on the lateral malleolus; the stable arm, along the head of the fibula; and the moving arm, along the fifth metatarsal. The physiotherapist stabilized the affected leg by one hand and moved ankle passively to maximum possible dorsiflexion by the other hand [27].

### Ankle Joint Proprioception

The ankle joint proprioception was measured with the patient sitting on the edge of bed with eyes closed. The electrogoniometer was connected to the longitudinal axis of the tibia and the fifth metatarsal. The physiotherapist slowly and randomly moved the ankle to one of the following angles: 5° plantar flexion, 15° plantar flexion, or 15° dorsiflexion angles. The examiner then held the ankle in that position for 5 seconds and asked the patient to note the ankle position. The ankle was moved passively to the starting position. The ankle was moved again to the desired position, and the patient was asked to report the position. The difference between the starting and the patient-reported position was recorded as an error value. These steps were repeated for 3 times, and the average error (in degree) over those 3 repetitions was considered as a reconstruction error of that angle [28]. The same procedure was performed for all angles with 1-minute rest interval, and the average error was recorded for each angle.

### Statistical Analysis

SPSS version 22 (SPSS Inc) was used for the data analysis. The normal distribution was analyzed using the Shapiro-Wilk test. The *t* test was used to examine the differences between 2 groups. Mixed model univariate analysis of variance (ANOVA) was used to analyze the effect of spasticity level of ankle plantar flexor muscles on the postural sway indicators in open- and closed-eyes conditions. The relationship between the severity of spasticity with the ABC scores, ankle proprioception, passive ROM, and TUG test were analyzed with Spearman rank correlation. The statistical significance was defined at α<.05.

## Results

Data collection and statistical analysis are complete. The interpretation of results is underway. The demographic characteristics of the participants will be calculated and provided. Descriptive results for all clinical and posturography measures will be reported and illustrated in the tables. The differences between 2 groups on the outcome measures will be analyzed and reported. The results of correlation coefficients between spasticity severity and clinical measures will be calculated and reported. We expect results to be published in winter 2020.

## Discussion

This study protocol will compare the static and dynamic balance in patients with stroke with high and low levels of plantar flexor spasticity. The results of this study would be relevant to clinicians addressing the challenges of spasticity and neurorehabilitation in patients after stroke.

There are a few studies focusing on the role of severity of spasticity on the poststroke balance dysfunction. Depression, gait asymmetry, and spasticity are 3 independent factors for predicting falls in patients with stroke [15]. Spasticity is a contributing factor to gait asymmetry [29,30]. It follows that the spasticity may be considered as one of the main predictors of falling, impairment in independent walking, and disability. Therefore, considering the role of lower limb spasticity in balance and gait dysfunctions of patients after stroke, the findings of this study will be important for both clinicians and patients to manage the plantar flexor spasticity, improve the balance, and enhance the walking ability and quality of life of the patients with stroke.

We have hypothesized that the balance dysfunction will be greater in the patients with high ankle plantar flexor spasticity than in the patients with low ankle plantar flexor spasticity. Further, the balance dysfunction will be greater with eyes closed than with eyes open. Additionally, the proprioception is reduced in the patients with stroke [31]. This impairment in proprioception is greater in the patients with higher ankle plantar flexor spasticity [32]. Consequently, we expect that the balance confidence will be lower in the group with high ankle plantar flexor spasticity. If the role of spasticity level in motor function of the patients with stroke is verified, it can help physiotherapists take necessary interventions to manage the ankle plantar flexor spasticity and improve proprioception. Such interventions can reduce the risk of falling and improve balance and mobility.

This study used a single-force platform. Thus, posturography measure was a net characteristic of both affected (paretic) and nonaffected feet. With 2 force plates (1 for each limb), the posturography characteristics of the affected foot and the nonaffected foot can be assessed for the 2 groups. These results can be then compared with those of neurologically healthy subjects.

The patients with high ankle plantar flexor spasticity will demonstrate greater static and dynamic balance dysfunctions than those of the patients with low spasticity, particularly, with eyes closed. The findings of this study will have implications for practice and research in the treatment of balance dysfunctions in patients with ankle plantar flexor spasticity after stroke.

## Abbreviations

ABC: Activities-Specific Balance Confidence
ANOVA: analysis of variance
MAS: Modified Ashworth Scale
MMAS: Modified Modified Ashworth Scale
ROM: range of motion
TUG: Timed Up and Go
